# Training modalities for elder sarcopenic obesity: a systematic review and network meta-analysis

**DOI:** 10.3389/fnut.2025.1537291

**Published:** 2025-02-19

**Authors:** Hao Qiu, Wanxia Zheng, Xi Zhou, Qianrong Liu, Xuehong Zhao

**Affiliations:** ^1^Department of Critical Care Medicine & Department of Emergency Medicine, Xiangyang Central Hospital, Affiliated Hospital of Hubei University of Arts and Science, Xiangyang, Hubei, China; ^2^Department of Rehabilitation Medicine, Xiangyang Central Hospital, Affiliated Hospital of Hubei University of Arts and Science, Xiangyang, Hubei, China; ^3^Medical College, Hubei University of Arts and Science, Xiangyang, Hubei, China

**Keywords:** aging, exercise training, multicomponent training, obesity, sarcopenia, sarcopenic obesity

## Abstract

**Introduction:**

Sarcopenic obesity (SO) is a condition characterized by the coexistence of age-related obesity and sarcopenia. This systematic review and network meta-analysis (NMA) aimed to compare the effects of different training modalities, such as aerobic training (AT), resistance training (RT), combined resistance with AT (CT), and multicomponent training (MCT) on body composition, muscle strength, and physical performance in elderly patients with SO.

**Methods:**

We electronically searched randomized controlled trials, published from inception to March 2024 in PubMed, Embase, Cochrane Library, Web of Science and Scopus. Effect estimates were presented as mean differences (MD) or Standard Mean Difference (SMD) with 95% confidence interval (CI). The comprehensive effects of all treatments were ranked by the surface under the cumulative ranking (SUCRA) probabilities.

**Results:**

14 trials enrolling 955 participants were included. The body fat percentage (BFP) in MCT (MD= −6.37, 95% CI: −8.67, −4.07), CT (MD = −2.08, 95% CI: −4.00, −0.16), and RT (MD = −1.85, 95% CI: −3.25, −0.44) was significantly lower than in the normal control group, with MCT showing superior effects compared to CT and RT. Furthermore, only MCT significantly improved fat-free mass (FFM; MD = 5.21, 95% CI:1.51, 8.91), as well as in body mass index (BMI; MD = 0.74, 95% CI:0.08, 1.40). In addition, handgrip strength (HGS) significantly improved under both MCT (SMD = 0.87, 95% CI: 0.19, 1.5) and RT(SMD = 0.84, 95% CI: 0.43, 1.25). The performance on the 30s chair stand test also yielded better outcomes for MCT (MD = 3.10, 95% CI: 1.33, 4 0.86), CT(MD = 2 0.50, 95% CI: 0.18, 5.18), and RT(MD = 3.91, 95% CI: 2.30, 5.52) when compared to the control group. Lastly, gait speed was enhanced by both MCT (MD = 0.35, 95% CI: 0.30, 0.41) and CT(MD = 0.14, 95% CI: 0.06, 0.21). The ranking results indicated that MCT was superior to other training modalities in enhancing body composition and gait speed. In contrast, RT appears to be more advantageous in the 30-second chair standing test and in improving HGS.

**Conclusion:**

MCT outperformed other training modalities in improving body composition and gait speed. RT was more beneficial for the 30-second chair standing test and enhancing HGS. These findings provide valuable insights for clinicians and researchers to customize exercise prescriptions for older patients with SO.

**Systematic review registration:**

http://www.crd.york.ac.uk/PROSPERO/display_record.asp?ID=CRD42024544962.

## 1 Introduction

Sarcopenic obesity (SO) is a complex clinical and functional condition characterized by the concurrent presence of obesity, marked by excessive fat mass (FM), and sarcopenia (1, 2). Sarcopenia, defined by diminished skeletal muscle mass and impaired physical function, is acknowledged as a geriatric syndrome with its incidence signifcantly increasing as individuals age (3, 4). Given the global trend toward population aging (5), the persistent prevalence of obesity (6), and the increasingly refined diagnostic criteria for sarcopenia, the incidence of sarcopenic obesity is rising at an alarming rate (7–9). SO is associated with numerous adverse health outcomes, including frailty, fractures, cardiovascular diseases, cancer, and increased hospitalization rates and mortality, thereby imposing a substantial social and economic burden (10, 11). Consequently, the prevention and treatment of SO in elderly individuals have become critical priorities in both research and clinical practice.

Although multiple pathogenesis mechanisms are involved, physical inactivity is a common and critical pathophysiological factor contributing to both sarcopenia and obesity (12, 13). Therefore, exercise training (ET) is widely acknowledged as one of the most cost-effective strategies for the prevention and management of SO (14–16). ET encompasses a variety of modalities, including resistance training (RT) (17), aerobic training (AT) (18, 19), flexibility (20, 21), and balance/gait training (22). Different exercise regimens induce distinct physiological adaptations. Specifically, AT is generally considered the most effective mode for enhancing cardiorespiratory fitness and reducing fat mass (19, 21, 23), while RT significantly improves muscle mass and strength (24, 25). Balance exercises contribute to improved stabilit, thereby reducing the risk of falls and enhancing daily functional performance (26). These improvements collectively enhance overall health and patient outcomes. However, not all exercise modalities uniformly enhance every aspect of muscle strength and physical performance in older adults with SO (27). Elderly patients with SO frequently experience multiple concurrent impairments, such as reduced muscle strength, diminished cardiorespiratory fitness, and impaired balance (28, 29). Consequently, exercise strategies that can address all these aspects within a single session may be more efficient for potential exercisers (30–32). This has resulted in the development of multicomponent training (MCT), which integrates at least three types of training, such as strength, aerobic, balance, and/or flexibility training (33–36). Research has demonstrated that MCT positively influences muscle mass, strength, gait, balance, and cardiorespiratory fitness in elderly individuals (33, 35, 37). Various position statements and consensus guidelines for physical activity in healthy older adults advocate for a multimodal exercise prescription (38, 39). Several recent clinical studies have highlighted the benefits of the MCT program on physical fitness in older adults with SO (40–44). However, it remains unclear whether MCT is more effective than single exercise protocols in treating SO.

Several systematic reviews have investigated the effects of exercise interventions on improving body fat, muscle mass, muscle strength, and physical performance in individuals with SO (27, 45–52). However, these traditional meta-analyses predominantly focus on comparing data from specific training modes against non-exercise control groups. While they can validate the efficacy of particular interventions, they lack a comprehensive analysis and comparison of various exercise modalities, thus failing to provide a clear understanding of the relative merits of different approaches. To date, no study has systematically compared these intervention types or comprehensively ranked various exercise interventions based on their efficacy in preventing and treating SO. Consequently, the optimal exercise type prescription for preventing or treating SO remains uncertain.

This study aimed to conduct a network meta-analysis (NMA) to comprehensively compare and evaluate the relative efficacy of different exercise modalities, such as AT, RT, combined training (CT, combined RT with AT), and MCT, on body composition and physical performance in elderly individuals with SO. This analysis will also provide evidence-based research support for formulating exercise prescriptions and bridge the gap between research and clinical implementation in exercise prescriptions for SO.

## 2 Materials and methods

This NMA was performed following the PRISMA 2020 principles (53) and registered in PROSPERO (CRD42024544962).

### 2.1 Search strategy

We electronically searched the studies published from inception to March 2024 in the following electronic databases: PubMed, Embase, Cochrane Library, Web of Science, and Scopus. We conducted the search on Boolean logic using the following terms: (“sarcopenia” OR “sarcopenic”) AND (“obese” OR “obesity” OR “overweight”) AND (“exercise” OR “training” OR “physical activity”). All retrieved references were exported to the EndNote (version X9) software to exclude duplicates as well as facilitate the initial screening and article selection as per the criteria. The detailed search strategy for each database is mentioned in Supplementary Appendix 1. Additionally, we also scanned the references of the included articles to segregate those who met the inclusion criteria. Two independent reviewers (WX and QR) screened the articles’ titles and abstracts to exclude ineligible articles. Subsequently, the chosen full-text articles were further screened to ensure that they met the inclusion criteria. Any disagreements between the reviewers were resolved either by discussion or the input of a third assessor (XH).

### 2.2 Eligibility criteria

The inclusion criteria were as follows: randomized and/or controlled clinical trials; participants ≥60 years with SO; and intervention: any ET mode alone without incorporating other treatments was one of the interven-tion arms. ET mode included AT, RT, RT, CT, and MCT. The control group included either educational or psychological intervention or no intervention. Outcome measurements encompassed at least one aspect of body composition [e.g., body fat percentage [BFP], body mass index [BMI], or fat-free mass (53)], muscle strength (assessed in upper or lower extremities), or physical performance (measured by gait speed and the 30-s chair stand test).

The exclusion criteria were as follows: animal studies, case studies, cross-sectional or retrospective studies, and review articles; sarcopenia or obesity alone as well as osteosarcopenic obesity; other medical complications, such as cancer, liver cirrhosis, or renal failure; absence of a standard control group; exercise intervention with other supportive treatments (e.g., nutritional supplements, medication, or calorie-restricted diet); and outcome measurements without any one of the body composition aspects, muscle function, or physical performance.

### 2.3 Data extraction

Two reviewers (WX and X) independently extracted data using predefined data forms based on the Cochrane Handbook. Additionally, each study provided the following data: author details, publication year, criteria for diagnosis of SO, sample sizes, the exercise intervention details, the body composition [bio-impedance analysis (BIA) or dual-energy X-ray absorptiometry (DXA)] measurement techniques, and participants’ baseline characteristics in each arm. For outcome measures, the number of patients, mean values, and standard deviations were extracted. When raw data was missing, we contacted the authors by email to request the data. Disagreements were resolved by discussion between the two reviewers and the trial information review.

### 2.4 Study risk of bias assessment

The Cochrane Risk of Bias Tool RoB 2.0 was employed to assess the quality (54). The risk of bias in the studies was assessed by two independent authors (H and QR) as per the recommendations in the Cochrane Handbook for Systematic Reviews of Interventions (55). Moreover, disagreements were resolved by a third reviewer (XH). Review Manager (RevMan) version 5.4.1 software was used to summarize the results of the bias risk assessment. Potential publication bias was investigated through visual inspection of funnel plots using the criterion of symmetry (56) and was assessed by Egger’s regression asymmetry test.

### 2.5 Statistical analysis

All analyses were performed using Stata version 15.1 (Stata Corp LP, College Station, TX, United States), and *p* values <0.05 indicated statistical significance. Mean difference (MD) was used to estimate the changes in baseline and post-intervention outcome measurements. In the case of variable study units, we calculated the standard mean difference (SMD) to determine the effect size. The MD or SMD was calculated with a 95% confidence interval (CI) for determining efficacy outcomes. The heterogeneity across trial results was tested with Cochran’s Q test and I^2^ statistic (57). If the Q test *p* > 0.1 and I^2^ 50%, indicating low inconsistency between the results of individual trials, the pooled effect was calculated using a fixed-effects model; If I^2^ > 50% or *p* < 0.1, considered as high heterogeneity between studies and a random-effects model was used. Then subgroup analysis on intervention modalities and intervention duration were conducted in outcomes that had a sufficient number of studies and the results were subsequently presented in forest plots.

NMAs were conducted using the Stata 15.1 “mvmeta” and “network” packages based on a frequentist analysis framework for all outcome measures. Initially stored in a long format (one record per treatment per study), the raw data were imported into an augmented format. Firstly, a network diagram with nodes and lines was constructed to summarize the comparative relationships among exercise interventions and controls. If a closed loop connecting different interventions existed, we used an inconsistency model and the node-splitting method for global and local inconsistencies, respectively. The results were subsequently presented in forest plots and league tables. Once the comparative effectiveness of the treatments had been evaluated, the treatments were ranked to identify their superiority. The interventions’ relative ranking was estimated based on the surface under the cumulative ranking curve (SUCRA), ranging from 0 to 100%. A higher SUCRA value denoted that the therapy was in the top rank (56, 58).

## 3 Results

### 3.1 Study screening process and results

**Figure 1:**
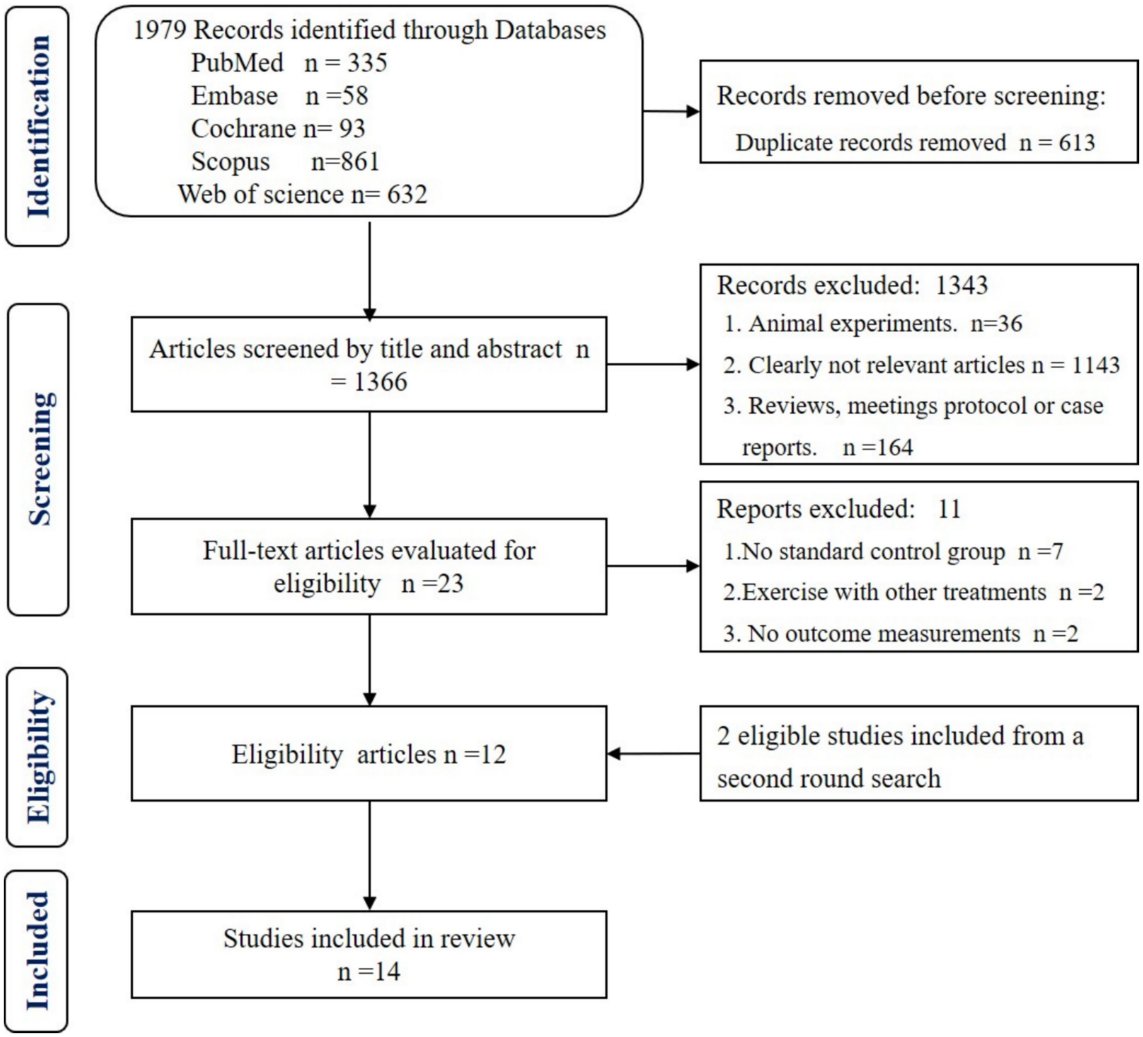
Flowchart of study selection following the PRISMA guidelines.

The initial electronic search identified 1979 studies. After duplicate removal, 1,366 records were included for title and abstract screening. After the exclusion of irrelevant titles and abstracts, the full texts of 23 studies were further screened. Consequently, 12 eligible RCTs were included in our systematic review. Following an updated search conducted via Google Scholar and references in August 2024, two additional eligible papers were identified and included. Thus, 14 studies (40–42, 44, 59–68) were included in the final analysis. Detailed information is provided in the PRISMA flow diagram (Figure 1).

### 3.2 Study characteristics

A total of 955 participants with SO and involvement in the exercise program were found in 14 studies published between 2016 and 2023. The characteristics of the included studies are displayed in Table 1.

**Table 1:** Characteristics of included studies.

Study (Year)	Nation	Sample Size	Intervention				Sex (F/M)^2^	Age(yr)	BMI (kg/m^2^)	Body fat (%)	Diagnosis criteria^3^	
			Type^1^	Session	Times/week	Duration (weeks)					Sarcopenia	Obesity
Ferhi (2023)	France	20	MCT	60 min	2	24	NA	74.1 ± 3.7	35.8 ± 2.1	43.0 ± 3.5	HGF < 17 N GS < 1.0 m/s	BMI > 30 kg/m^2^
		20	Control	daily activities			NA	76.6 ± 5.6	35.8 ± 2.7	42.1 ± 4.5		
Magtouf (2023)	France	25	MCT	60 min	3	16	NA	76.3 ± 3.5	34.5 ± 4.0	39.0 ± 4.5	HGF < 17 N GS < 1.0 m/s	BMI > 30 kg/m^2^
		25	Control	Non-intervention			NA	75.9 ± 5.4	34.7 ± 2.3	40.0 ± 4.3		
Chiu (2018)	China (Taiwan)	33	RT	60 min	2	12	22/14	79.64 ± 7.36	25.15 ± 3.75	42.07 ± 6.0	TSM/BW < 37.15% M < 32.26% F	BFP ≥ 29% M ≥ 40%
		31	Control	Non-intervention			13/21	80.15 ± 8.26	24.85 ± 3.01	40.29 ± 6.87		
Liao (2017)	China (Taiwan)	25	RT	55 min	3	12	F	66.39 ± 4.49	27.32 ± 3.33	43.09 ± 5.14	TSM/BW´100% < 27.6%	BFP >30%
		21	Control	Non-intervention				68.42 ± 5.86	28.19 ± 3.27	44.82 ± 5.52		
Liao (2018)	China (Taiwan)	33	RT	55 min	3	12	F	66.39 ± 4.49	27.32 ± 3.33	43.09 ± 5.14	TSM /Height^2^ < 7.15 kg/m^2^	BFP >30%
		23	Control	Non-intervention				68.42 ± 5.86	28.19 ± 3.27	44.82 ± 5.52		
Jung (2022)	Korea	14	MCT	45 ~ 75 min	3	12	F	75.36 ± 4.50	22.50 ± 1.75	35.10 ± 3.13	ASM /Height^2^ ≤ 5.4 kg/m^2^	BFP >32%
		14	Control	Usual care				74.64 ± 5.77	22.58 ± 1.69	35.33 ± 3.18		
Chen (2017)	China (Taiwan)	15	RT	60 min	2	8	13/2	68.9 ± 4.4	28.3 ± 4.4	39.7 ± 5.6	ASM/BW ≤ 32.5% M ≤ 25.7% F	BMI ≥ 25 kg/m^2^
		15	AT	60 min	2		14/1	69.3 ± 3.0	26.8 ± 3.8	40.0 ± 4.4		
		15	CT	60 min	2		11/4	68.5 ± 2.7	27.2 ± 2.9	39.7 ± 5.8		
		15	Control	Non-intervention			13/2	68.6 ± 3.1	29.0 ± 3.9	39.8 ± 4.5		
Huang (2017)	China (Taiwan)	18	RT	55 min	3	12	F	68.89 ± 4.91	27.31 ± 3.74	41.66 ± 7.65	TSM/BW´ < 27.6% F	BFP >30%
		17	Control	Health education				69.53 ± 5.09	28.96 ± 3.49	42.39 ± 6.07		
Park (2017)	Korea	25	CT	50–80 min	3 RT 5 AT	24	F	73.5 ± 7.1	27.0 ± 1.4	41.0 ± 3.6	ASM/BW´ < 25.1%	BMI ≥ 25 kg/m^2^
		25	Control	usual activities				74.7 ± 5.1	27.6 ± 2.0	40.4 ± 3.6		
Vasconcelos (2016)	Brazil	14	RT	60 min	2	10	F	72 ± 4.6	32 ± 2.3	NA	HGF ≤ 21 kg	BMI ≥ 30 kg/m^2^
		14	Control	non-intervention				72 ± 3.6	33 ± 2.9	NA		
Kim (2016)	Japan	35	CT	60 min	2	12	F	81.4 ± 4.3	25.1 ± 2.5	37.0 ± 4.1	ASM/Height^2^ < 5.67 kg/m^2^/HGF <17 kg / GS < 1.0 m/s	BFP ≥ 32%
		34	Control	Health education				81.1 ± 5.1	25.3 ± 2.8	38.5 ± 3.3		
Gadelha (2016)	Brazil	69	RT	NA	3	24	F	66.79 ± 5.40	27.10 ± 3.99	39.85 ± 6.27	Residual values	
		64	Control	non-intervention				67.27 ± 5.04	29.09 ± 5.08	39.8 ± 6.51		
WANG (2019)	China	20	RT	30 min	2	8	11/9	65.1 ± 3.4	29.0 ± 3.02	39.8 ± 4.05	FNIH standard	NA
		20	AT	30 min	2		10/10	64.2 ± 3.0	26.5 ± 3.82	41.2 ± 3.42		
		20	CT	30 min	2		12/8	63.6 ± 5.2	26.5 ± 3.82	39.7 ± 5.83		
		20	Control	non-intervention			10/10	64.1 ± 2.8	29.0 ± 3.02	39.8 ± 4.05		
Marcos-Pardo (2020)	Brazil	114	MCT	60 min	3	12	NA	68.03 ± 4.03	31.13 ± 4.15	NA	EWGSOP standard	BMI >25 kg/m^2^
		102	Control	non-intervention			NA		28.68 ± 3.04	NA		

1 AT, aerobic training; RT, resistance training; CT, combined resistance with aerobic training; MCT, multiple component training.

2 F, Female; M, Male; NA, not applicable.

3 BMI, Body mass index; BFP, Body fat percentage; GS, Gait Speed; HGF, Handgrip force; TSM, Total skeletal muscle mass; ASM, appendicular skeletal mass.

BW, Body weight. Residual values: The residual values of a regression equation that predicts appendicular FFM based on height (meters) and fat mass (kilograms) (46, 54). FNIH standard: Standard from the Foundation for the National Institutes of Health Biomarkers Consortium Sarcopenia (106). EWGSOP standard: Sarcopenia criteria from the European Working Group on Sarcopenia in Older People (EWGSOP) (4).

### 3.3 Risk of bias assessment

**Figure 2:**
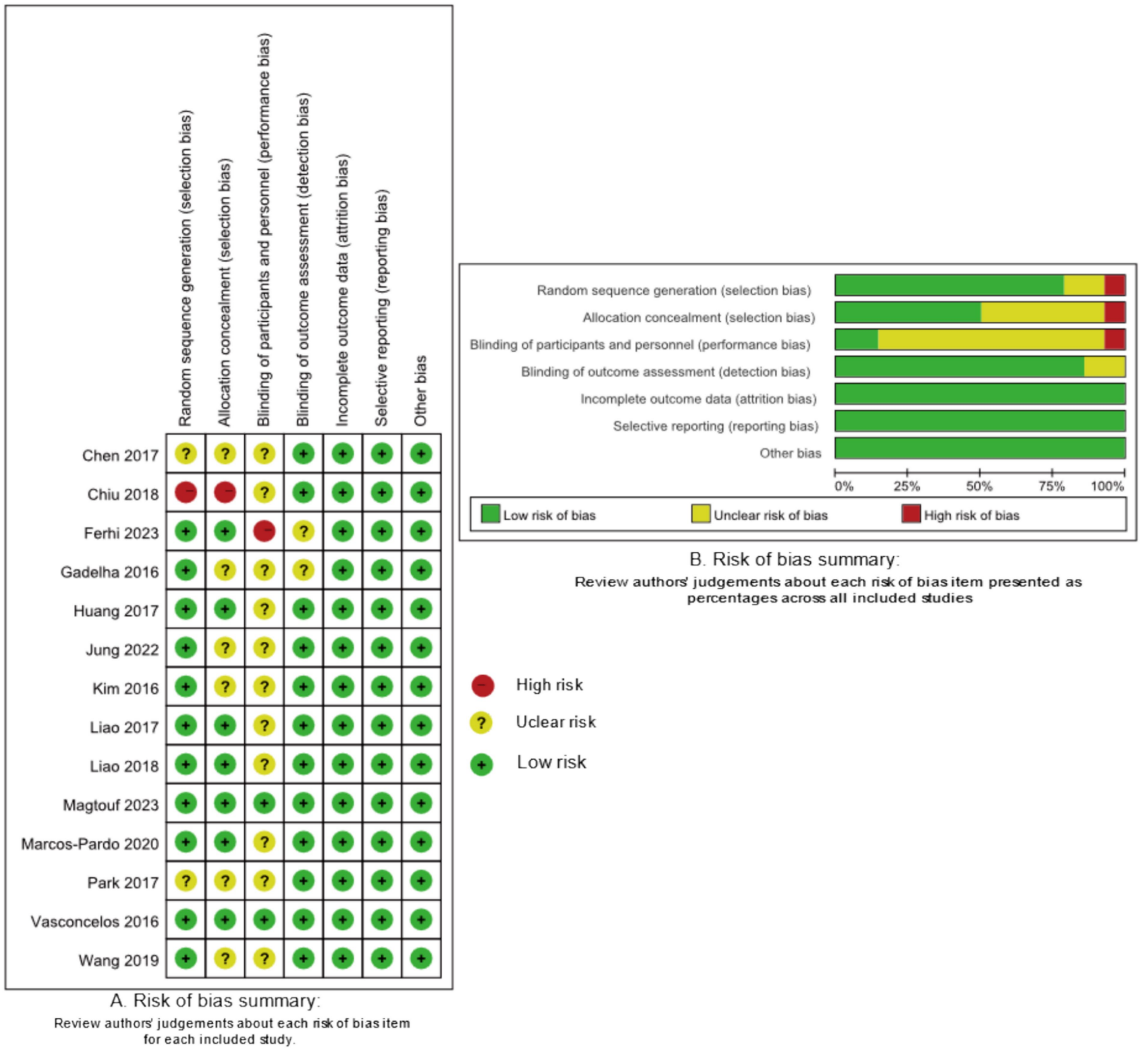
Risk-of-bias assessment according to the revised Cochrane risk-of-bias tool.

As seen in Figure 2, the risk of bias was high, unclear, and low in 2, 10, and 2 studies, respectively. Since one trial adopted the nonrandomized health interventions’ evaluations, it was judged as a high risk of bias for selection and performance. Moreover, one trial was assessed as high risk of bias due to its single-blind program. Additionally, 10 trials were assessed as having unclear risk of bias owing to insufficient data on the random sequence generation, allocation concealment, or the outcome measurement. The detail annotation for clarity is presented in Supplemental Appendix 2.

Overall, we did not find any publication bias across the included studies. All outcome measurements’ funnel plots were visually symmetrically distributed around the mean estimated treatment effect. The *p*-values for Egger’s test were: 0.076 for BFP, 0.275 for BMI, 1.31 for FFM, 0.977 for handgrip strength (HGS), 0.321 for 30-s chair stand test, and 0.99 for gait speed (Supplementary Appendix 3).

### 3.4 NMA

**Figure 3:**
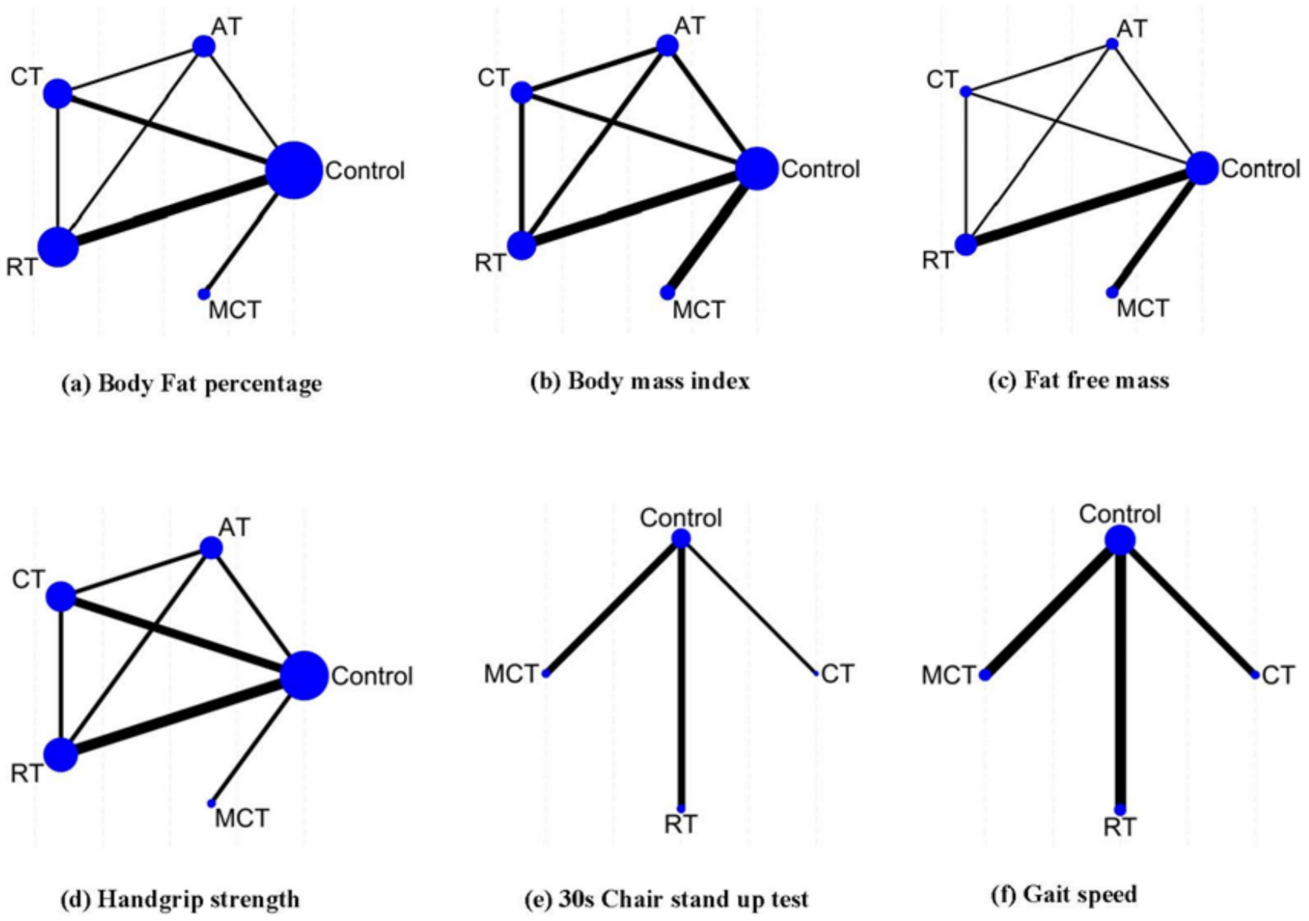
Network geometry summary. The size of the edges is proportional to the number of studies, and the size of the nodes is proportional to the number of each intervention. AT, aerobic training; RT, resistance training; CT, combined resistance with aerobic training; MCT, multiple component training.

A total of four different interventions and control arms were included in our NMA. The primary outcomes’ network geometry is shown in Figure 3. Among the 14 eligible studies, most studies reported at least two indicators each: 12 studies focused on BFP, 8 assessed BMI, 7 examined FFM, 9 evaluated HGS, 5 investigated the 30-s chair stand test,and 8 examined gait speed. The characteristics of the intervention methods for each outcomes are displayed in Supplementary Appendix 4.

The NMA-based inconsistency test showed no statistically significant differences in global inconsistency (BFP: *p* = 0.7268; BMI: *p* = 0.6657; FFM: *p* = 0.9597, and HGS: *p* = 0.3634). The closed-loop network evaluation revealed no statistically significant differences in inconsistency between direct and indirect outcomes (Supplementary Appendix 5).

#### 3.4.1 NMA on BFP

**Figure 4:**
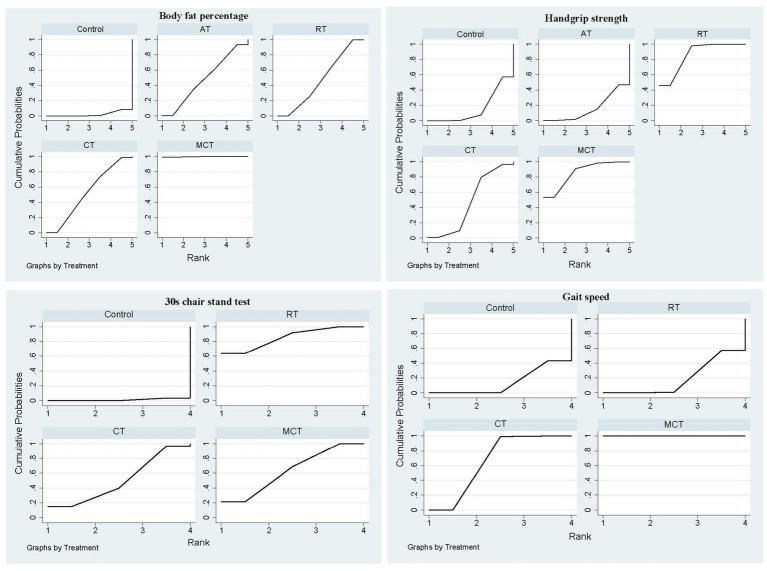
The primary outcome’s cumulative ranking probability plot. AT, Aerobic training; CT, Combined resistance with aerobic training; MCT, Multicomponent training; RT, Resistance training.

We included 12 studies and 711 subjects. The results showed that MCT [MD = −6.37, 95% CI-8.67, −4.07)], CT [(MD = −2.08, 95% CI (−4.00, −0.16)], and RT [MD = −1.85, 95% CI (−3.25, −0.44)] were superior to the control group. Additionally, MCT was significantly better than RT [MD = −4.52, 95% CI (−7.17, −1.88)] and CT [MD = −4.29, 95% CI (−7.25, −1.33)] groups, respectively. However, no significant difference was observed between the AT and the control group or between other exercise interventions (Table 2). The ranked results showed that MCT was superior to CT and RT; MCT was the most effective technique in reducing BFP in older adults with SO (Figure 4; Table 3).

**Table 2:** League table depicting NMA findings.

Body fat percentage	MCT				
	**−4.29 (−7.25, −1.33)**	CT			
	−**4.52 (−7.17, −1.88)**	−0.23 (−2.42, 1.95)	RT		
	**−4.44 (−7.87, −1.01)**	−0.15(−2.93, 2.62)	0.08 (−2.57, 2.73)	AT	
	**−6.37 (−8.67, −4.07)**	−2.08 (−4.00, −0.16)	−1.85 (−3.25, −0.44)	−1.93 (−4.49, 0.63)	Control
Body mass index	MCT				
	−0.51 (−2.02, 1.00)	CT			
	−0.56 (−1.53, 0.42)	−0.05 (−1.45, 1.35)	RT		
	−0.68 (−2.43, 1.06)	−0.18 (−1.80, 1.45)	−0.13 (−1.78, 1.52)	AT	
	−0.74 (−1.40, −0.08)	−0.23 (−1.59, 1.13)	−0.18 (−0.93, 0.56)	−0.05 (−1.67, 1.57)	Control
Fat free mass	MCT				
	4.18 (−2.79, 11.15)	CT			
	4.86 (−0.03, 9.74)	0.68 (−5.24, 6.59)	RT		
	4.68 (−2.21, 11.57)	0.50 (−5.99, 6.99)	−0.18 (−6.00, 5.65)	AT	
	**5.21 (1.51, 8.91)**	1.03 (−4.88, 6.93)	0.35 (−2.83, 3.53)	0.53 (−5.28, 6.34)	Control
Handgrip strength	MCT				
	0.57 (−0.25, 1.38)	CT			
	0.03 (−0.76, 0.82)	−0.54 (−1.07, 0.00)	RT		
	0.91 (−0.01, 1.82)	0.34 (−0.31, 0.98)	**0.87 (0.23, 1.51)**	AT	
	**0.87 (0.19, 1.55)**	0.30 (−0.15, 0.76)	**0.84 (0.43, 1.25)**	−0.03 (−0.65, 0.58)	Control
30-s chair stand test (repetitions)	MCT				
	0.60 (−2.62, 3.81)	CT			
	−0.82 (−3.26, 1.63)	−1.41 (−4.54, 1.72)	RT		
	**3.10 (1.33, 4.86)**	2.50 (−0.18, 5.18)	**3.91 (2.30, 5.52)**	Control	
Gait speed	MCT				
	**0.22 (0.12, 0.31)**	CT			
	**0.35 (0.25, 0.44)**	**0.13 (0.02, 0.23)**	RT		
	**0.35 (0.30, 0.41)**	**0.14 (0.06, 0.21)**	0.01 (−0.07, 0.08)	Control	

The table displays the MD/SMD with a 95% confidence interval. Significant results are highlighted in bold. AT, Aerobic training; CT, Combined resistance with aerobic training; MCT, Multicomponent training; RT, Resistance training.

**Table 3:** The primary outcome rankings for different exercise techniques.

Training type	Body fat percentage			Handgrip strength			30s chair stand test			Gait speed		
	SUCRA	PrBest	Mean rank	SUCRA	PrBest	Meanrank	SUCRA	PrBest	Meanrank	SUCRA	PrBest	Mean rank
Control	2.2	0.0	4.9	16.2	0	4.4	1.1	0.0	4.0	0.7	0.0	4.0
AT	47.9	0.6	3.1	15.9	0.1	4.4	–	–	–	–	–	–
RT	47.2	0	3.1	85.9	45.9	1.6	85.1	63.7	1.4	46.9	0.1	2.6
CT	52.9	0.2	2.9	46.6	0.8	3.1	50.5	15.0	2.5	52.5	0.4	2.4
MCT	99.8	99.3	1.0	85.4	53.2	1.6	63.3	21.3	2.1	99.8	99.5	1.0

AT, Aerobic training; CT, Combined resistance with aerobic training; MCT, Multicomponent training; RT, Resistance training.

The ranked results demonstrated that MCT outperformed CT and RT, establishing MCT as the most effective technique for reducing BFP in older adults with SO (Figure 4; Table 3). Furthermore, subgroup analysis based on intervention duration revealed that MCT consistently ranked as the optimal treatment for BFP across both short-term (≤12 weeks) and long-term (>12 weeks) intervention periods (Supplementary Appendix 6).

#### 3.4.2 NMA on BMI

We included eight studies and 642 subjects. The NMA results showed that only MCT intervention was superior to the control group [MD = 0.74, 95% CI (0.08, 1.40)]. Nonetheless, no significant difference was observed between the other interventions and the control group or between interventions (*p* > 0.05; Table 2).

#### 3.4.3 NMA on FFM

A total of seven studies and 315 subjects were utilized. Our results showed that only MCT intervention was better than the control group [MD = 5.21, 95% CI (1.51, 8.91)]. However, there was no significant difference between the other interventions and the control group or between interventions (Table 2).

#### 3.4.4 NMA on HGS

We used nine studies and 515 subjects. HGS with RT [SMD = 0.84, 95% CI (0.43, 1.25)] and MCT 0.87 (0.19, 1.55) [MD = 0.87, 95% CI (0.19, 1.55)] were superior to the control group. No significant differences were observed between the other interventions and the control group or between interventions (*p* > 0.05; Table 2). The ranked results showed that RT was the most effective intervention in improving HGS in older adults with SO (Figure 4; Table 3).

#### 3.4.5 NMA on 30-s chair stand test (repetitions)

A total of five studies and 408 participants were utilized (Table 1). Although 30s chair stand repetitions improved in MCT [MD = 3.10, 95% CI (1.33, 4.86)] and RT [MD = 3.91, 95% CI (2.30, 5.52)] when compared with control groups; however, the MCT and RT values were similar [MD = −0.82, 95% CI (−3.26, 1.63)]. There was no significant difference between the other interventions and the control group or between interventions (Table 2). The ranked results showed that while RT was superior to MCT, it was the most effective modality in improving 30s chair stand repetitions in older adults with SO (Figure 4; Table 3).

#### 3.4.6 NMA on gait speed

A total of eight studies and 555 subjects were involved. Our results showed that MCT [MD = 0.35, 95% CI (0.30, 0.41)] and CT [MD = 0.14, 95%CI (0.06, 0.21)] exerted better effects than the control group; however, MCT was superior to CT [MD = 0.22, 95% CI (0.12, 0.31)]. No significant differences were observed between the RT and the control group [MD = 0.01, 95% CI (−0.0.07, 0.08), Table 2]. While MCT was superior to CT, MCT was the most effective technique in improving gait speed in older adults with SO (Figure 4; Table 3).

Furthermore, subgroup analysis based on intervention duration revealed that MCT was the sole modality that significantly enhanced speed in the short-term intervention group. However, in the long-term intervention group, MCT demonstrated a superior effect compared to CT (Supplementary Appendix 6).

## 4 Discussion

This systematic review and NMA on exercise interventions for elders adults with SO included data from 14 clinical trials involving 955 participants. To our knowledge, this is the first NMA to explore the relative efficacy of different exercise modes on body composition and physical performance in older adults with SO. Our results confirm the beneficial effects of exercise interventions on body composition and physical performance, and highlighting MCT may as the most promising exercice strategy for addressing SO.

### 4.1 NMA on body composition

The body composition of SO patients is characterized by increased adipose tissue and decreased muscle mass. Optimal results require simultaneously increasing skeletal muscle mass and reducing body fat (69). The latest study from Liu et al. indicated that a 12-week CT intervention improved muscle strength and cardiopulmonary fitness in older adults with sarcopenia, while body composition remained unchanged (70). Our results showed that both RT and CT significantly reduced BFP, but neither of them decreased BMI nor improved FFM. RT is considered the most effective intervention for improving muscle mass; however, its efficacy is compromised in individuals with obesity (71). These findings align with the previous meta-analyses (27, 47, 49). Notably, MCT not only significantly reduced BFP more effectively than RT and CT but also increased FFM simultaneously. This is consistent with prior studies showing that MCT improves almost all body composition parameters in middle-aged and older women, particularly in overweight participants (72, 73). Therefore, MCT may be the optimal exercise strategy for improving body composition in older individuals with SO. A considerable body of literature has established that low-grade inflammation is a key factor in the progressive loss of muscle mass and increased fat accumulation (74–76). Extensive research has demonstrated that physical exercise exhibits anti-inflammatory properties (77), with different exercise modalities exerting varying effects on inflammatory biomarkers (74). Jung et al. reported that after 12 weeks of MCT, elevated high-sensitivity CRP (hs-CRP) and IL-6 levels in SO patients were significantly reduced (44). Gargallo et al. found that MCT decreased inflammatory status by downregulating CRP in obese subjects, whereas RT did not have this effect (78). Chen et al. also identified MCT as the most effective exercise modality for ameliorating IL-6, TNF-*α*, and IL-10, while RT had the least effect compared to other exercise types (74). It is widely accepted that significant positive correlations were observed between reductions in body fat and the effect sizes of hs-CRP, TNF-α, and IL-10 (73–78). These findings suggest that the reduction in body fat and enhancement of FFM following MCT may be attributed to improvements in inflammatory markers (44).

### 4.2 NMA of HGS and 30-s chair stand test

HGS serves as a primary indicator of upper limb strength, and diminished HGS is a robust and independent predictor of sarcopenia (3, 4, 79). In line with previous studies (78, 80, 81), our findings demonstrate that RT significantly enhances HGS. Moreover, our NMA revealed that MCT has a comparable positive effect on HGS to that of RT, which aligns with the findings of Labott et al. (82). Several mechanisms are likely responsible for the substantial improvement in HGS following RT. These include alterations in muscle fiber type composition (83, 84), activation and proliferation of satellite cells (85), increased rates of mitochondrial protein synthesis (86), and enhanced motor unit recruitment (87, 88).

Chair stand tests are widely acknowledged as a reliable indicator of lower limb strength (89) and are frequently utilized in the diagnosis of sarcopenia (90). Consistent with the HGS findings, the 30-s chair stand test also exhibited significant improvements after both RT and MCT interventions, thereby validating the efficacy of these methods in enhancing lower extremity strength. Consequently, our results align with the studies by Poli et al. (91, 92) and the systematic review by Labata-Lezaun et al. (34), which collectively demonstrated that MCT significantly increases strength in both upper and lower extremities. In this study, the intervention duration for the RT group in literature related to HGS and the 30-s chair stand test ranged from 8 to 12 weeks, whereas that for the MCT group ranged from 12 to 24 weeks. However, the ranked results indicated that RT performed better than MCT in both measures. This finding is consistent with prior research that has demonstrated the efficacy of RT in enhancing muscle strength, even over relatively brief periods (93).

### 4.3 NMA on gait speed

Gait speed is the most widely utilized assessment tool for evaluating physical performance in individuals with sarcopenia (3, 4, 79). Consistent with the meta-analyses of Hsu et al. (49) and Zhuang et al. (45), our study demonstrated that CT significantly improved gait speed, whereas RT did not yield significant improvements. Furthermore, we found that multicomponent training (MCT) also significantly enhanced gait speed in sarcopenic older (SO) patients, with MCT showing superior efficacy compared to CT. Cadore et al. conducted a systematic review of exercise interventions for gait ability in frail elderly individuals and concluded that MCT can significantly improve gait performance, while RT alone has limited efficacy (94). The study by Wang et al. demonstrated that a two-week MCT intervention significantly improved gait speed in very old inpatients with sarcopenia (95). Collectively, these studies highlight the advantages of MCT in improving gait speed, which aligns with our findings. Reduced gait speed has been associated with age-related declines in lower extremity muscle strength, endurance, balance, motor control, and cognition (96–99). Additionally, walking speed has been shown to have an inverse relationship with the proportion of adipose tissue in the quadriceps muscle (80) and a positive correlation with with FFM (97). Previous research has shown that MCT is highly beneficial for reducing fat infiltration and enhancing muscle strength, endurance, and balance in older adults (100, 101), particularly in improving cognitive function (102). Our results further confirm that MCT is the only type of exercise that improves both muscle composition and function. Therefore, MCT demonstrates a significant advantage in enhancing gait speed compared to other forms of exercise, and we recommend MCT as the primary intervention for treating SO, particularly in individuals with pronounced weakness and physical performance impairments.

### 4.4 Strengths and limitations

This study exhibits several significant strengths. Firstly, to the best of our knowledge, this is the first NMA that systematically compared and quantitatively summarized the efficacy of various exercise modalities on SO. Secondly, we adhered strictly to a rigorous inclusion criterion by exclusively incorporating randomized controlled trials (RCTs), which are regarded as the gold standard in clinical research.

Our NMA had several limitations that merit attention. Firstly, the number of studies included was limited to 14. In particular, with only two RCTs involving 35 participant focusing on AT. This relatively small sample size restricts the generalizability of the findings to broader populations of older adults with SO. Given that 80.75% of the study participants were female, the applicability of the treatment across all genders requires further investigation. Therefore, future research should prioritize conducting more RCTs with larger and more diverse sample sizes, including a broader representation of genders. Secondly, among the included studies, only two utilized multi-arm designs, neither of which involved MCT. Consequently, many effect size estimates relied heavily on indirect comparisons. Additionally, this NMA focused exclusively on the effects of different exercise modalities and did not include dose–response analysis.

### 4.5 Reasons for the heterogeneity

Publication bias and small sample size bias were assessed using Egger’s test and visually examined via a funnel plot of effect size (ES) relative to standard error. No evidence of publication bias was detected across all outcomes in the included studies, indicating a minimal likelihood of publication bias or small sample effects. Heterogeneity among studies was assessed using the Q-test and the I^2^ statistic. The results indicated substantial heterogeneity (I^2^>75% and *p* < 0.05) across all outcomes. Subgroup analyses based on intervention modalities revealed that, apart from the MCT group, there was no significant heterogeneity within the other subgroups (Supplementary Appendix 7).This suggests that the primary source of statistical heterogeneity may be attributed to variations in exercise modality. Given that MCT incorporates a diverse array of components, this inherently leads to variability in the combinations and sequences of training programs (31, 103–105). Any inconsistencies in the integration strategies for these modalities, the sequencing of its components, or the duration of each phase, can significantly impact the overall effectiveness of MCT. Therefore, the heterogeneity observed in the MCT group may stem from variations in practical implementation. Consequently, further research is imperative to identify the specific exercise parameters—including components, sequences, intensity, duration, and frequency—that optimize the benefits of MCT for patients with SO.

## 5 Conclusion

The current NMA demonstrated that MCT outperformed other exercise intervention models in enhancing body composition and gait speed. Moreover, RT showed a significant advantage in enhancing muscle strength, while MCT’s efficacy in strength improvement was comparable to that of RT. Given that MCT has been shown to significantly enhance both morphology and function in patients with SO, it appears to be the most optimal and efficacious exercise strategy for addressing this condition. However, due to the limited number of studies in this field, future research should prioritize conducting more high-quality RCTs to validate the positive effects of MCT on individuals with SO. Additionally, future investigations should aim to determine the optimal combination of exercise types and dosages for MCT programs to maximize their beneficial impacts on individuals with SO.

## Data Availability

The original contributions presented in the study are included in the article/Supplementary material, further inquiries can be directed to the corresponding author.
