# Supplementary material for: Training modalities for elder sarcopenic obesity: a systematic review and network meta-analysis
Source: Front Nutr. 2025 Feb 19;12:1537291. doi: 10.3389/fnut.2025.1537291 (PMC11879820; doi:10.3389/fnut.2025.1537291)
Supplement: Supplementary file 1 [file Data_Sheet_1.pdf]

Appendix 1. Search strategy

1. PubMed **Time: 25/12/2023 23:05:11**

#	Query	Search Details	Results
4	((((obesity[MeSH Terms]) OR (obese[Title/Abstract])) OR (overweight[Title/Abstract])) AND (((sarcopenia[MeSH Terms])) OR (sarcopenic[Title/Abstract]))) AND (((exercise[MeSH Terms]) OR (training[Title/Abstract])) OR (physical activity[Title/Abstract]))	("obesity"[MeSH Terms] OR "obese"[Title/Abstract] OR "overweight"[Title/Abstract]) AND ("sarcopenia"[MeSH Terms] OR "sarcopenic"[Title/Abstract]) AND ("exercise"[MeSH Terms] OR "training"[Title/Abstract] OR "physical activity"[Title/Abstract])	335
3	((exercise[MeSH Terms]) OR (training[Title/Abstract])) OR (physical activity[Title/Abstract])	"exercise"[MeSH Terms] OR "training"[Title/Abstract] OR "physical activity"[Title/Abstract]	874,136
2	((sarcopenia[MeSH Terms])) OR (sarcopenic[Title/Abstract])	"sarcopenia"[MeSH Terms] OR "sarcopenic"[Title/Abstract]	11,482
1	((obesity[MeSH Terms]) OR (obese[Title/Abstract])) OR (overweight[Title/Abstract])	"obesity"[MeSH Terms] OR "obese"[Title/Abstract] OR "overweight"[Title/Abstract]	352,990

2. Cochrane Library **Date Run: 15/03/2024 23:05:11**

ID	Searches	Results
#1	MeSH descriptor: [Sarcopenia] explode all trees	884
#2	(sarcopenic):ti,ab,kw	432
#3	#1 OR #2	1146
#4	MeSH descriptor: [Obesity] explode all trees	21150
#5	(obese):ti,ab,kw	27270
#6	(overweight):ti,ab,kw	22011
#7	#4 OR #5 OR #6	45598
#8	MeSH descriptor: [Exercise] explode all trees	38461
#9	(training):ti,ab,kw	129075
#10	(physical activity):ti,ab,kw	58263
#11	#8 OR #9 OR #10	187870
#12	#3 AND #7 AND #11	93

3. Web of Science Search Strategy (v0.1)

Date Run   Sat Mar 16 2024 02:01:35 GMT+0800

Database: Web of Science Core Collection

#	Search Query	Results
1	(TS=(sarcopenia)) OR TI=(sarcopenic)	25779
2	((TS=(obesity)) OR TS=(obese)) OR TS=(overweight)	569254
3	((TS=(exercise)) OR TI=(training)) OR TI=(physical activity)	922561
4	#1 AND #2 AND #3	867
5	#1 AND #2 AND #3 and Review Article (Exclude – Document Types)	632

4. **Embase** Last update 2024-3-15

#	Query	Last Results
4	#1 AND #2 AND #3	58
3	' exercise '/exp/mj OR training:ab, ti OR physical activity: ab, ti	389954
2	'obesity'/exp/mj OR obese: ab,ti OR overweight:ab,ti	94674
1	'sarcopenia'/exp/mj OR sarcopenic: ab,ti	14516

5. Scopus

Query	Results
TITLE-ABS-KEY (("sarcopenia" OR "sarcopenic") AND ("obese" OR "obesity" OR "overweight") AND ("exercise" OR "training" OR "physical activity") .) AND (EXCLUDE (DOCTYPE , "re") OR EXCLUDE (DOCTYPE , "ch") OR EXCLUDE (DOCTYPE , "ed") OR EXCLUDE (DOCTYPE , "le") OR EXCLUDE (DOCTYPE , "no") OR EXCLUDE (DOCTYPE , "er") OR EXCLUDE (DOCTYPE , "bk"))	861

Appendix 2. Risk of bias assessment of included studies using the revised Cochrane risk-of-bias tool

Study		Random sequence generation (Selection bias)	Allocation concealment (Selection bias)	Blinding of participants and personnel (Performance bias)	Blinding of outcome, assessment (Detection bias)	Incomplete outcome/data (Attrition bias)	Selective reporting, (Reporting bias)	Other bias
Chen 2017	Support for Judgement	No information	No information	No information	Quote: The staff conducted the trial measurements were blinded to treatment assignment.	No missing outcome data	All the pre-specified outcomes have been reported	No obvious other sources of bias
	Judgement	Unclear risk	Unclear risk	Unclear risk	Low risk	Low risk	Low risk	Low risk
Chiu 2018	Support for judgement	Nonrandomized	Nonrandomized	No information	The outcome measurement is not likely to be influenced	Missing data have been imputed using appropriate methods.	All the pre-specified outcomes have been reported	No obvious other sources of bias
	Judgement	High risk	High risk	Unclear risk	Low risk	Low risk	Low risk	Low risk
Ferhi 2023	Support for Judgement	Computer-generated	Central allocation	Incomplete blinding	No information	No missing outcome data	All the pre-specified outcomes have been reported	No obvious other sources of bias
	Judgement	Low risk	Low risk	High risk	Unclear risk	Low risk	Low risk	Low risk
Gadelha 2016	Support for Judgement	Computer-generated	No information	No information	No information	No missing outcome data	All the pre-specified outcomes have been reported	No obvious other sources of bias
	Judgement	Low risk	Unclear risk	Unclear risk	Unclear risk	Low risk	Low risk	Low risk
Huang 2017	Support for Judgement	A random number table	Sealed envelope	No information	Quote: the assessors was blinded to which group was participated	No missing outcome data	All the pre-specified outcomes have been reported	No obvious other sources of bias
	Judgement	Low risk	Low risk	Unclear risk	Low risk	Low risk	Low risk	Low risk
Jung 2022	Support for Judgement	Computer-generated	No information	No information	The outcome measurement is not likely to be influenced	No missing outcome data	All the pre-specified outcomes have been reported	No obvious other sources of bias
	Judgement	Low risk	Unclear risk	Unclear risk	Low risk	Low risk	Low risk	Low risk
Kim 2016	Support for Judgement	Computer-generated	No information	No information	The outcome measurement is not likely to be influenced	No missing outcome data	All the pre-specified outcomes have been reported	No obvious other sources of bias
	Judgement	Low risk	Unclear risk	Unclear risk	Low risk	Low risk	Low risk	Low risk

Appendix 2. Risk of bias assessment of included studies using the revised Cochrane risk-of-bias tool

**Study**		Random sequence generation (Selection bias)	Allocation concealment (Selection bias)	Blinding of participants and personnel (Performance bias)	Blinding of outcome, assessment (Detection bias)	Incomplete outcome/data (Attrition bias)	Selective reporting, (Reporting bias)	Other bias
Liao 2017	Support for Judgement	Computer-generated	Central allocation	No information	Quote: All outcome measures were assessed by a blinded examiner	No missing outcome data	All the pre-specified outcomes have been reported	No obvious other sources of bias
	Judgement	Low risk	Low risk	Unclear risk	Low risk	Low risk	Low risk	Low risk
Liao 2018	Support for Judgement	Computer-generated	Central allocation	No information	Quote: All outcome measures were assessed by a blinded examiner	No missing outcome data	All the pre-specified outcomes have been reported	No obvious other sources of bias
	Judgement	Low risk	Low risk	Unclear risk	Low risk	Low risk	Low risk	Low risk
Magtouf 2023	Support for judgement	Computer generated	Central allocation	Quote: TMP program were intentionally not disclosed to the participants	Quote: A blinded assessor conducted visits twice before and after the program	No missing outcome data	All the pre-specified outcomes have been reported	No obvious other sources of bias
	Judgement	Low risk	Low risk	Low risk	Low risk	Low risk	Low risk	Low risk
Marcos-Pardo 2020	Support for judgement	Computer generated	Central allocation	No information	The outcome measurement is not likely to be influenced	No missing outcome data	All the pre-specified outcomes have been reported	No obvious other sources of bias
	Judgement	Low risk	Low risk	Unclear risk	Low risk	Low risk	Low risk	Low risk
Park 2017	Support for judgement	No information	No information	No information	The outcome measurement is not likely to be influenced	No missing outcome data	All the pre-specified outcomes have been reported	No obvious other sources of bias
	Judgement	Unclear	Unclear	Unclear	Low	Low	Low risk	Low risk
Vasconcelos 2016	Support for judgement	Computer generated	Central allocation	No blinding，but the outcome is not likely to be influenced.	The assessors were blinded participant allocation	No missing outcome data	All the pre-specified outcomes have been reported	No obvious other sources of bias
	Judgement	Low risk	Low risk	Low risk	Low risk	Low risk	Low risk	Low risk
Wang 2019	Support for judgement	Computer generated	No information	No information	The outcome measurement is not likely to be influenced	No missing outcome data	All the pre-specified outcomes have been reported	No obvious other sources of bias
	Judgement	Low risk	Unclear risk	Unclear risk	Low risk	Low risk	Low risk	Low risk

Appendix 3. Funnel Plots

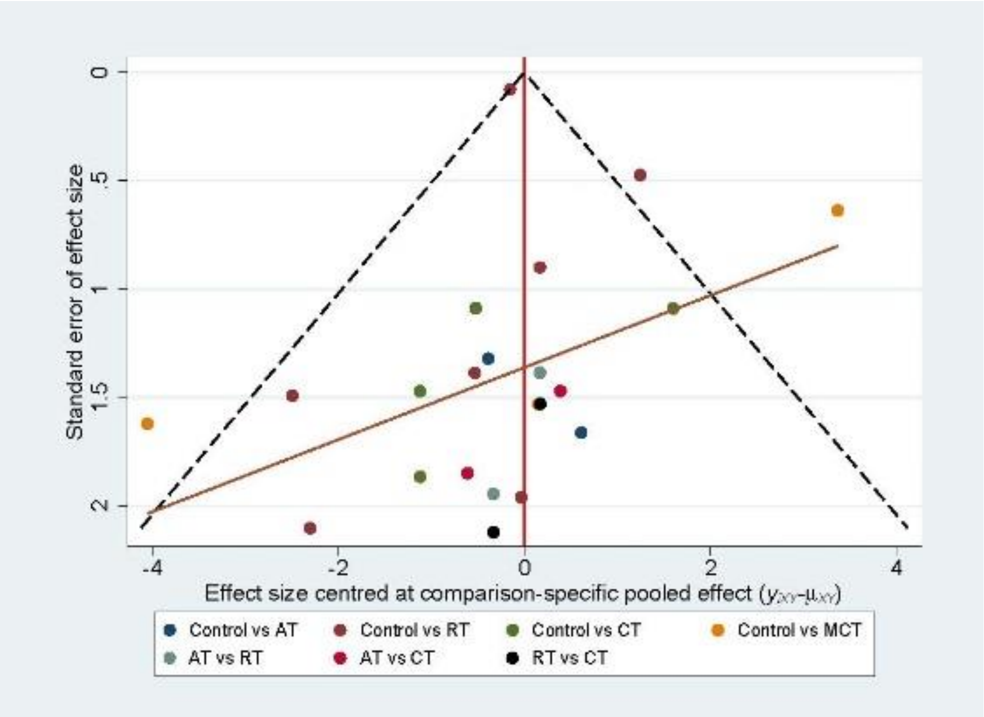

(a) **Body fat percentage (Egger's** test: P= 0.076)

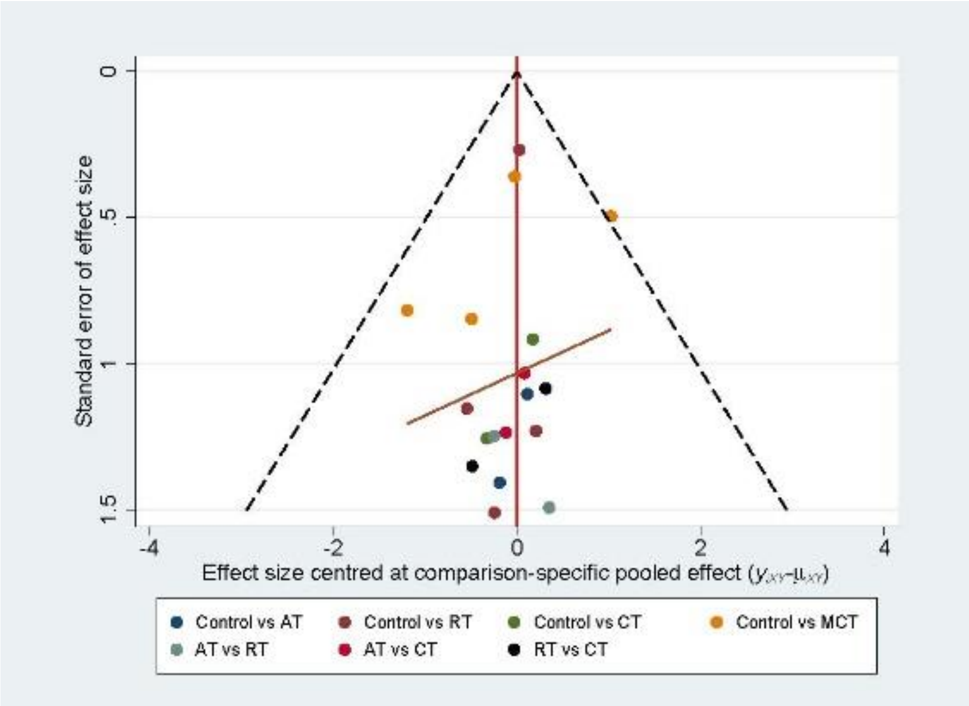

(b) **Body mass index (BMI) (**Egger's test: P= 0.275)

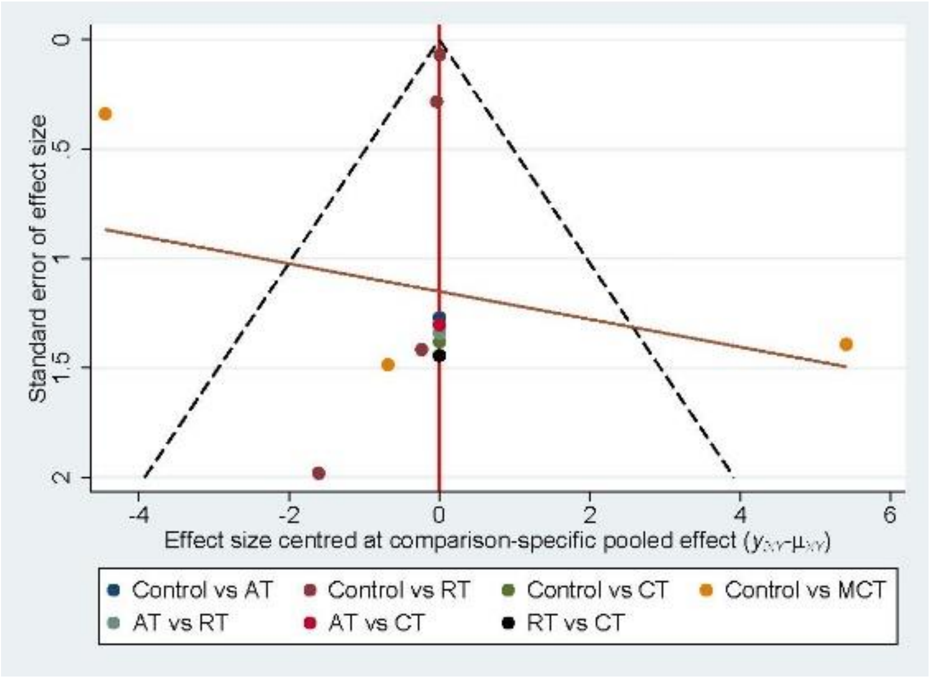

(c) **Fat free mass** (Egger's test: P= 1.31)

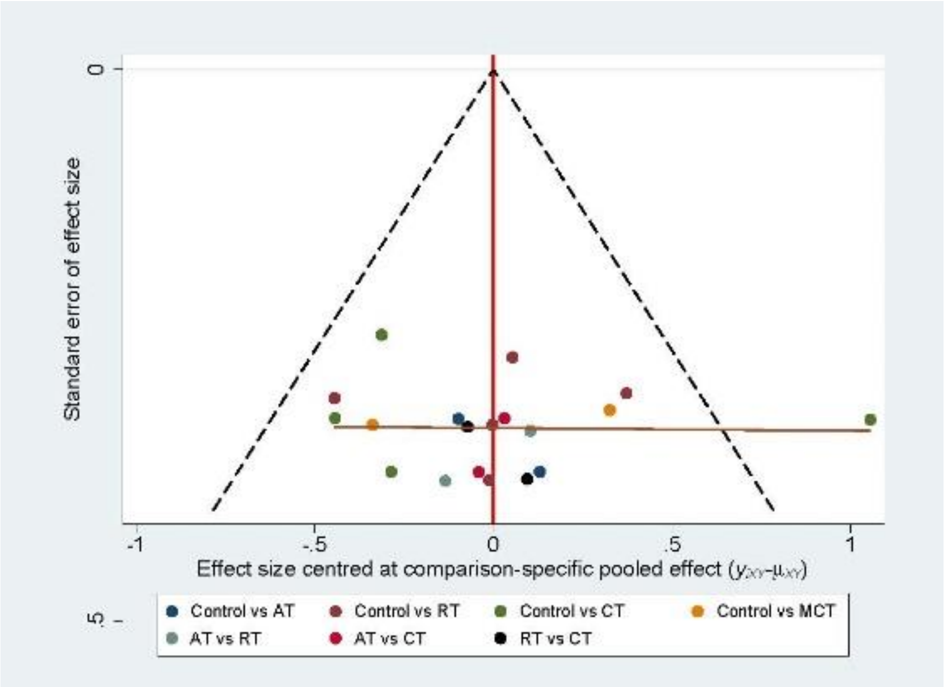

(d) **Handgrip strength** (Egger's test: P= 0.977)

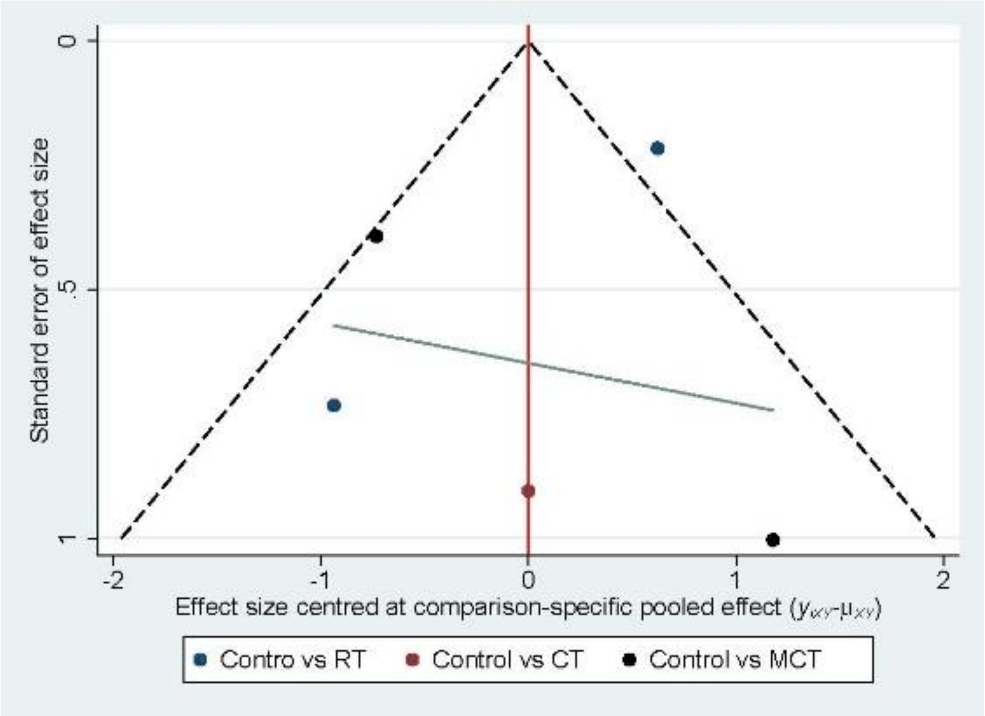

(e)30s chair stand test (repetitions) (Egger's test: P= 0.32

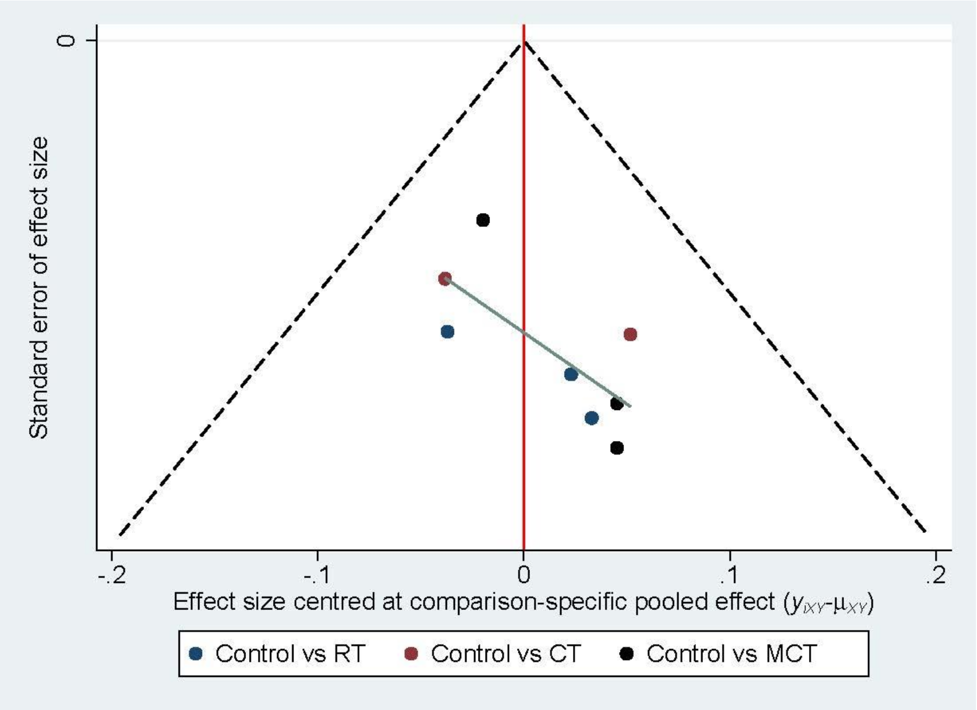

(f) Gait speed (Egger's test: P= 0.547)

Note: AT, Aerobic training; RT, Resistance training; CT, Combined resistance with aerobic training; MCT, Multicomponent training

Appendix 4 Characteristics of the Intervention

1.Body fat percentage

Mode	Reference	Session (min)	Duration (weeks)	Frequency / week	Total Sessions
MCT	Jung (2022)	45-75	12	3	36
	Ferhi (2023)	60	24	2	48
	Magtouf (2023)	60	16	3	48
CT	Chen (2017)	60	8	2	16
	WANG (2019)	30	8	2	16
	Kim（2016）	60	12	2	24
	Park (2017)	50-80	24	3	72
RT	Chen (2017)	60	8	2	16
	WANG (2019)	30	8	2	16
	Chiu (2018)	60	12	2	24
	Liao (2017)	55	12	3	36
	Liao (2018)	55	12	3	36
	Huang (2017)	55	12	3	36
	Gadelha (2016)	NA	24	3	72
AT	Chen (2017)	60	8	2	16
	WANG (2019)	30	8	2	16

2. Body mass index

Mode	Reference	Session (min)	Duration (weeks)	Frequency / week	Total Sessions
MCT	Jung (2022)	45-75	12	3	36
	Marcos-Pardo (2020)	60	12	3	36
	Ferhi (2023)	60	24	2	48
	Magtouf (2023)	60	16	3	48
CT	Chen (2017)	60	8	2	16
	WANG (2019)	30	8	2	16
RT	Chen (2017)	60	8	2	16
	WANG (2019)	30	8	2	16
	Huang (2017)	55	12	3	36
	Gadelha (2016)	NA	24	3	72
AT	Chen (2017)	60	8	2	16
	WANG (2019)	30	8	2	16

3. Fat free mass

Mode	Reference	Session (min)	Duration (weeks)	Frequency / week	Total Sessions
MCT	Jung (2022)	45-75 min	12	3	36
	Ferhi (2023)	60 min	24	2	48
	Magtouf (2023)	60 min	16	3	48
CT	Chen (2017)	60min	8	2	16
	Park (2017)	50-80min	24	3	72
RT	Chen (2017)	60min	8	2	16
	Liao (2017)	55min	12	3	36
	Liao (2018)	55min	12	3	36
	Huang (2017)	55min	12	3	36
AT	Chen (2017)	60min	8	2	16

4. HGS

Mode	Reference	Session (min)	Duration (weeks)	Frequency /week	Total Sessions
MCT	Ferhi (2023)	60	24	2	48
	Magtouf (2023)	60	16	3	48
CT	Chen (2017)	60	8	2	16
	WANG (2019)	30	8	2	16
	Kim（2016）	60	12	2	24
	Park (2017)	50-80	24	3	72
RT	Chen (2017)	60	8	2	16
	WANG (2019)	30	8	2	16
	Chiu (2018)	60	12	2	24
	Liao (2017)	55	12	3	36
	Liao (2018)	55	12	3	36
AT	Chen (2017)	60	8	2	16
	WANG (2019)	30	8	2	16

5. 30s chair stand test

Mode	Reference	Session (min)	Duration (weeks)	Frequency /week	Total Sessions
MCT	Ferhi (2023)	60	24	2	48
	Magtouf (2023)	60	16	3	48
CT	Chen (2017)	60	8	2	16
	WANG (2019)	30	8	2	16
	Kim（2016）	60	12	2	24
	Park (2017)	50-80	24	3	72
RT	Chen (2017)	60	8	2	16
	WANG (2019)	30	8	2	16
	Chiu (2018)	60	12	2	24
	Liao (2017)	55	12	3	36
	Liao (2018)	55	12	3	36
AT	Chen (2017)	60	8	2	16
	WANG (2019)	30	8	2	16

6. Gait speed

Mode	Reference	Session (min)	Duration (weeks)	Frequency /week	Total Sessions
MCT	Marcos-Pardo (2020)	60	12	3	36
	Ferhi (2023)	60	24	2	48
	Magtouf (2023)	60	16	3	48
CT	Kim（2016）	60	12	2	24
	Park (2017)	50-80	24	3	72
RT	Vasconcelos (2016)	60	10	2	20
	Liao (2017)	55	12	3	36
	Liao (2018)	55	12	3	36

Note: AT, Aerobic training; RT, Resistance training; CT, Combined resistance with aerobic training; MCT, Multicomponent training.

Appendix 5. Testing for inconsistency

1. Body fat percentage

(1) Testing for overall inconsistency

χ^2 =0.64, p =0.7268

No evidence for the existence of significant global inconsistency

(2) Node splitting test for inconsistency

Comparison of interventions	Direct		Indirect		Difference		P>z	tau
	Coefficient	Standard error	Coefficient	Standard error	Coefficient	Standard error		
Aerobic training *vs* Combined resistance with aerobic training	-0.742	1.603	2.544	3.575	-3.286	3.987	**0.41**	1.557
Aerobic training *vs* Control	2.253	1.531	0.918	2.743	1.335	3.147	**0.671**	1.578
Aerobic training *vs* Resistance training	0.268	1.628	-0.576	3.037	0.844	3.518	**0.81**	1.618
Combined resistance with aerobic training *vs* Control	2.028	1.056	2.871	3.393	0.844	3.518	**0.81**	1.618
Combined resistance with aerobic training *vs* Resistance training	1.009	1.699	0.433	1.576	1.442	2.318	**0.534**	1.6
Control *vs* Resistance training	-1.958	0.745	1.328	3.929	-3.286	3.987	**0.41**	1.557
Control vs Multiple component training	-6.370	1.174	-4.169	941.223	-2.201	941.224	**0.998**	1.513

2. Body mass index

(1) Testing for overall inconsistency

χ^2= 0.19, p = 0.6657

No evidence for the existence of significant global inconsistency

(2) Node splitting test for inconsistency

Comparison of interventions	Direct		Indirect		Difference		P>z	tau
	Coefficient	Standard error	Coefficient	Standard error	Coefficient	Standard error		
Aerobic training ***vs*** Resistance combined with aerobic training	-0.175	0.828	0.352	429.657	-0.527	429.658	**0.999**	0.337
Aerobic training ***vs*** Control	0.213	0.913	-0.705	1.955	0.918	2.123	**0.666**	0.401
Aerobic training ***vs*** Resistance training	-0.354	0.999	0.563	1.827	-0.918	2.123	**0.666**	0.401
Resistance combined with aerobic training vs Control	0.388	0.796	-0.529	1.904	0.918	2.123	**0.666**	0.401
Resistance combined with aerobic training vs Resistance training	-0.178	0.894	0.739	1.771	-0.918	2.123	**0.666**	0.401
Control vs Resistance training	-0.183	0.379	-0.838	484.035	0.655	484.035	**0.999**	0.337
Control ***vs*** Multiple component training	-0.738	0.338	-0.095	480.898	-0.644	480.898	**0.999**	0.337

3. Handgrip strength

(1) Testing for overall inconsistency

χ^2 **= 2.02, p= 0.3634**

No evidence for the existence of significant global inconsistency

(2) Node splitting test for inconsistency

Comparison of interventions	Direct		Indirect		Difference		P>z	tau
	Coefficient	Standard error	Coefficient	Standard error	Coefficient	Standard error		
Aerobic training ***vs*** Resistance combined with aerobic training	0.139	0.599	0.834	1.492	-0.695	1.607	0.665	0.775
Aerobic training ***vs*** Control	0.168	0.518	-1.801	0.986	1.97	1.113	0.077	0.648
Aerobic training ***vs*** Resistance training	0.933	0.541	3.012	1.232	-2.078	1.348	0.123	0.68
Resistance combined with aerobic training vs Control	-0.337	0.374	-2.416	1.295	2.078	1.348	0.123	0.68
Resistance combined with aerobic training vs Resistance training	0.798	0.596	1.432	0.755	-0.635	0.962	0.509	0.767
Control vs Resistance training	1.506	0.382	2.201	1.562	-0.695	1.607	0.665	0.775
Control ***vs*** Multiple component training	0.867	0.561	0.53	445.245	0.337	445.245	0.999	0.728

Appendix 6 Subgroup analysis of intervention duration

1.Body fat percentage

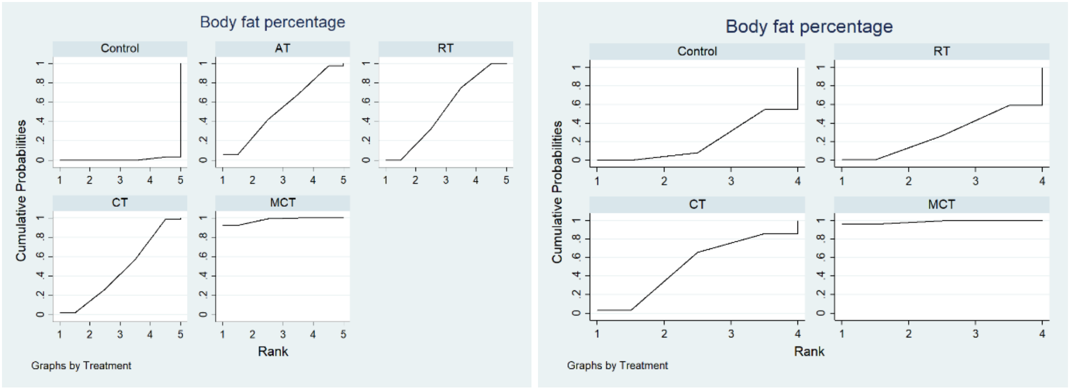

≤ 12weeks >12 weeks

Figure 1. The body fat percentage cumulative ranking probability plot

Table 1. The body fat percentage rankings for different exercise techniques

Training* Type	≤ 12 weeks			> 12weeks		
	SUCRA	PrBest	Mean Rank	SUCRA	PrBest	MeanRank
**Control**	0.9	0.0	5.0	21.0	0.0	3.4
**AT**	53.4	5.7	2.9	–	–	–
**RT**	51.9	0.1	2.9	**28.8**	**0.5**	**3.1**
**CT**	46.0	2.0	3.2	51.5	3.1	2.5
**MCT**	**97.8**	**92.2**	**1.1**	**98.7**	**96.4**	**1.0**

(A) ≤ 12weeks

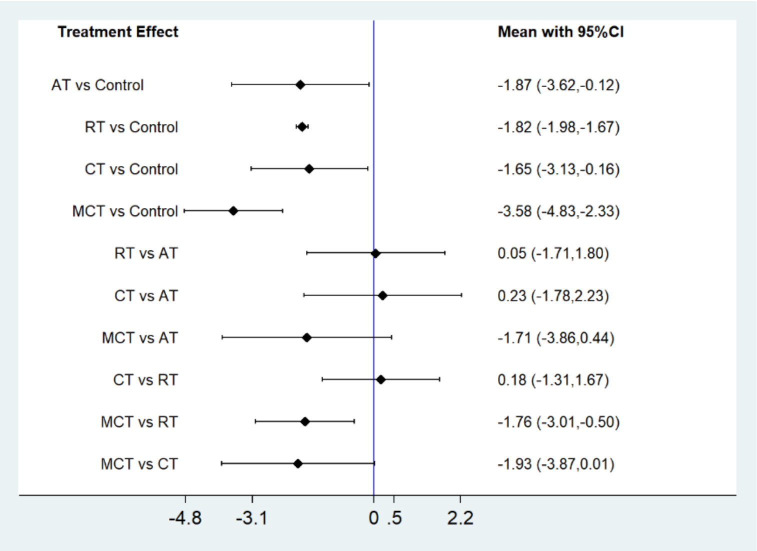

(B) >12 weeks

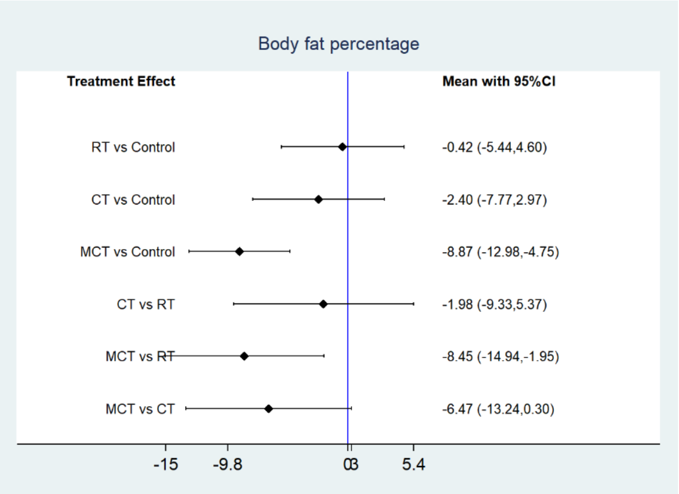

Figure 2. Forest plot summari zing effects of AT, RT, CT and MCT on changes of Body fat percentage at each intervention duration

2. Handgrip strength

(A) ≤ 12weeks

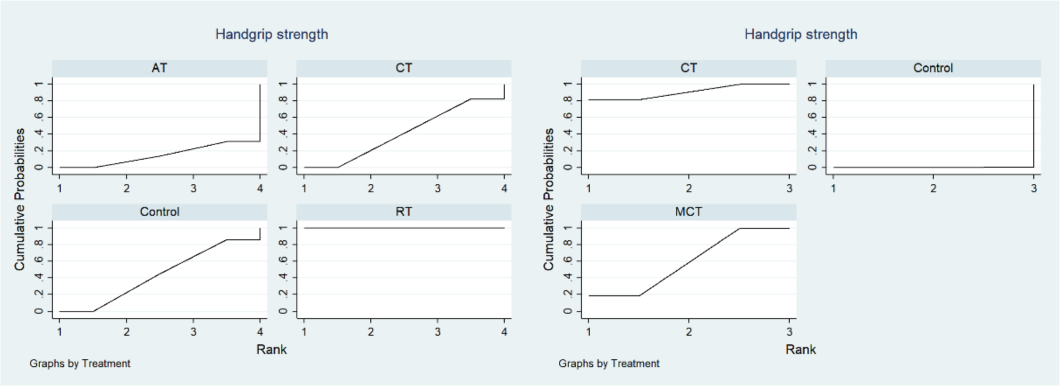

≤ 12weeks >12 weeks

Figure 3. The handgrip strength cumulative ranking probability plot

Table 2. The handgrip strength rankings for different exercise techniques

Training* Type	≤ 12 weeks			> 12weeks		
	SUCRA	PrBest	Mean Rank	SUCRA	PrBest	MeanRank
**Control**	43.6	0.0	2.7	0.3	0.0	3.0
**AT**	15.1	0.0	3.5	–	–	–
**RT**	100.0	100.0	1.0	–	–	–
**CT**	41.3	0.0	2.8	90.9	81.8	1.2
**MCT**	–	–	–	58.8	18.2	1.8

(A) ⩽ 12weeks

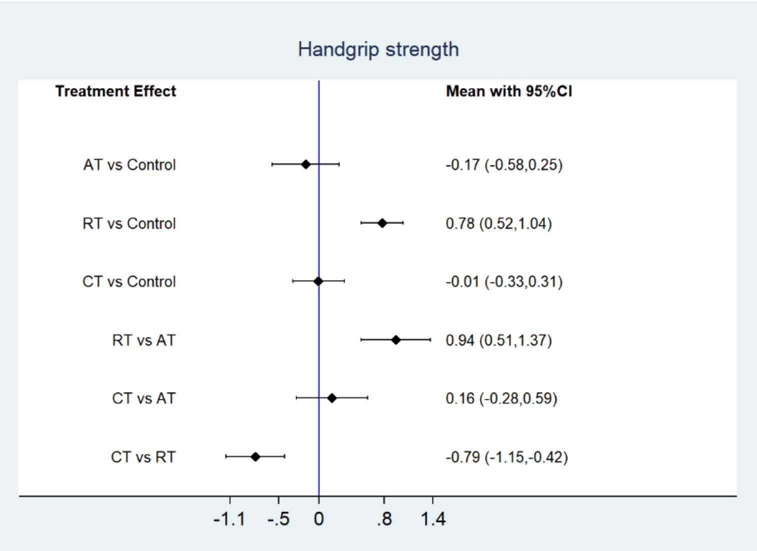

(B) >12 weeks

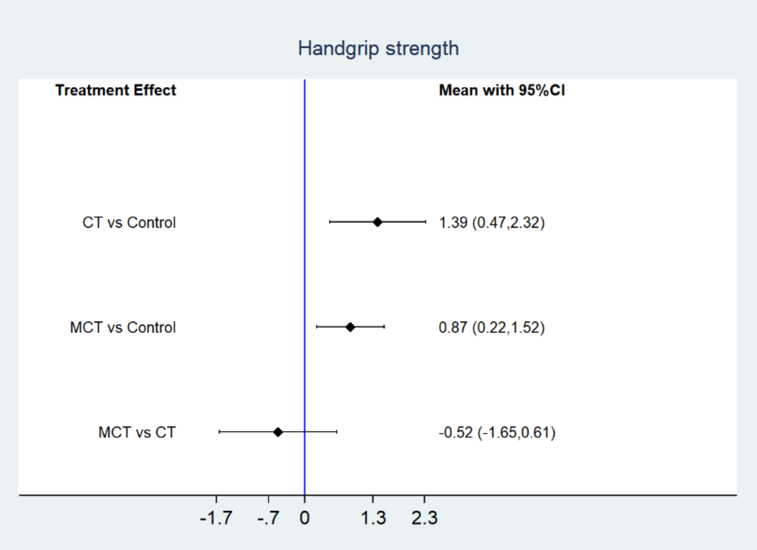

Figure 4. Forest plot summarizing effects of AT, RT, CT and MCT on changes of handgrip strength at each intervention duration

3.Gait speed

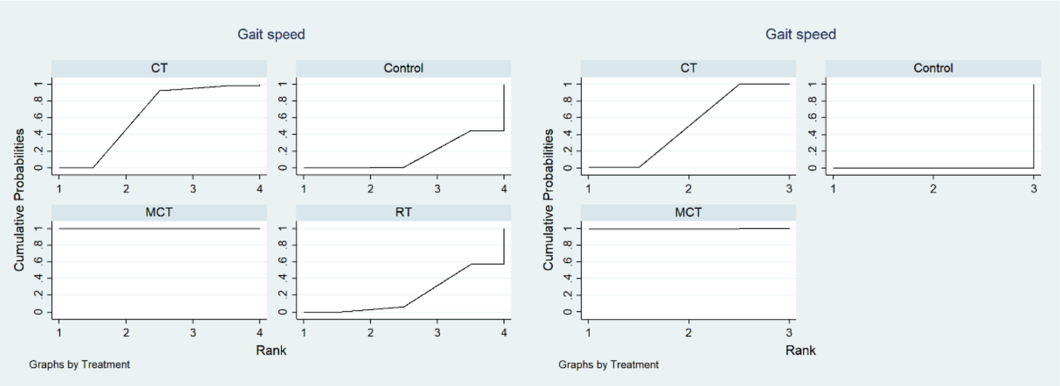

≤ 12weeks >12 weeks

Figure 5. The gait speed cumulative ranking probability plot

Table 3. The Gait speed rankings for different exercise techniques

Training* Type	≤ 12 weeks			> 12weeks		
	SUCRA	PrBest	Mean Rank	SUCRA	PrBest	MeanRank
**Control**	15.3	0.0	3.5	0.0	0.0	3.0
**AT**	–	–	–	–	–	–
**RT**	21.1	0.0	3.4	–	–	–
**CT**	63.6	0.0	2.1	50.2	0.4	2.0
**MCT**	100.0	100.0	1.0	99.8	99.6	1.0

(A) ≤ 12weeks

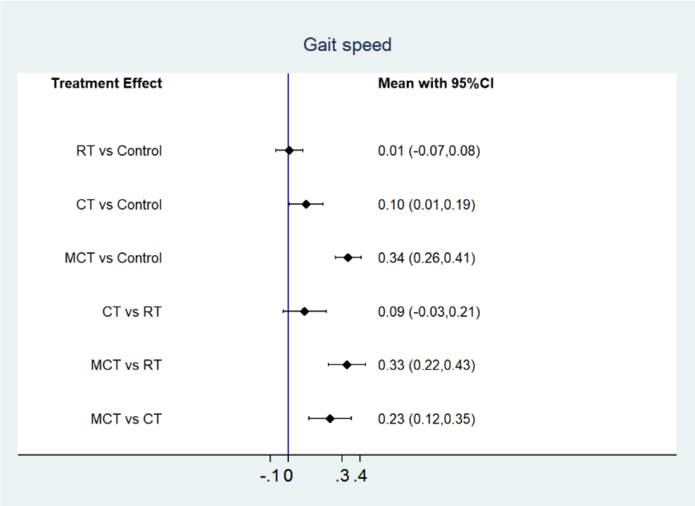

(B) >12 weeks

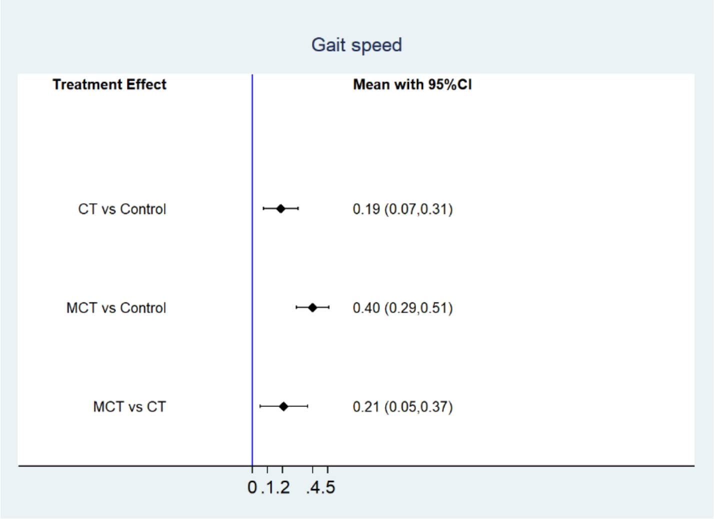

Figure 6. Forest plot summarizing effects of AT, RT, CT and MCT on changes of gait speed at each intervention duration

Note: AT, Aerobic training; RT, Resistance training; CT, Combined resistance with aerobic training; MCT, Multicomponent training.

Appendix 7. Pairwise meta-analyses
and subgroup by exercise mode

1. Body fat percentage

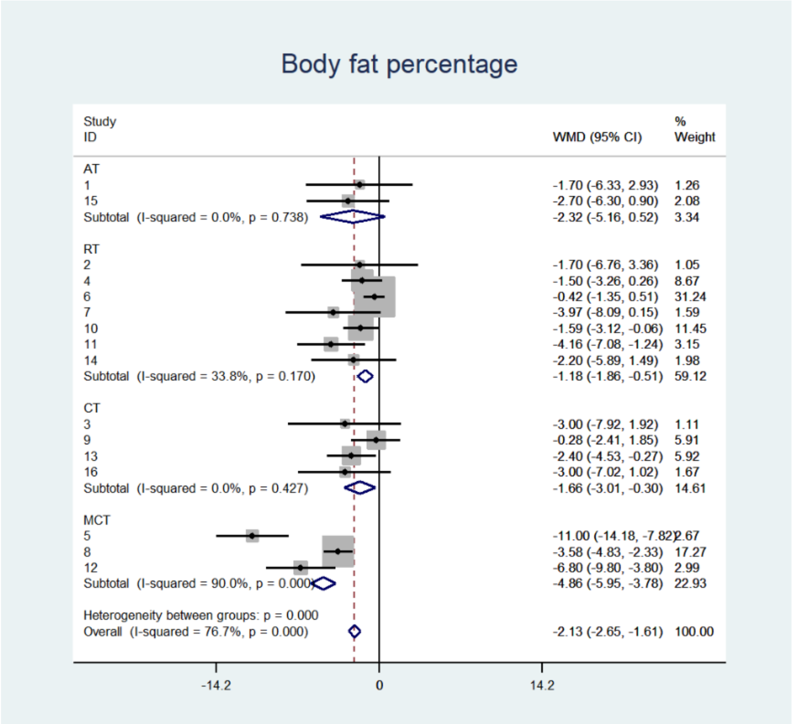

2. Body mass index

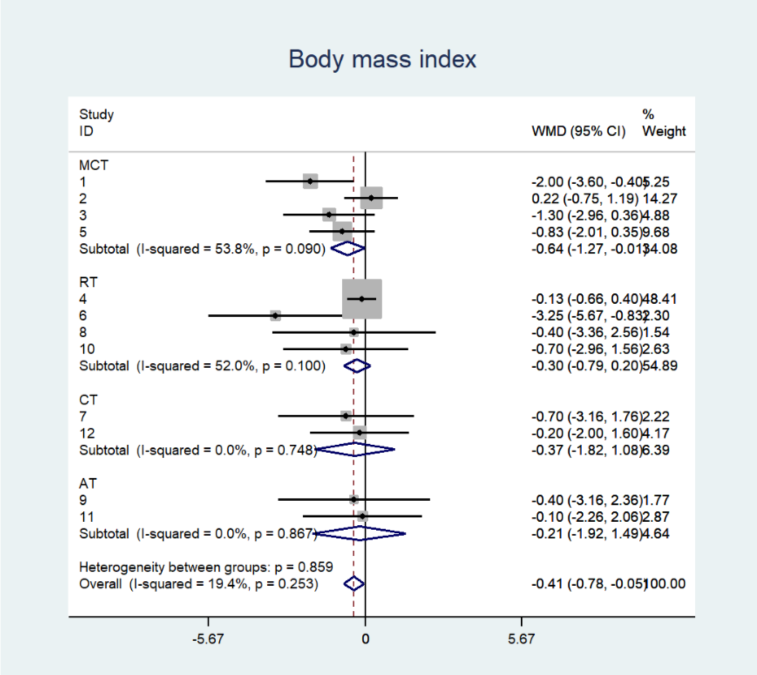

3. Fat free mass

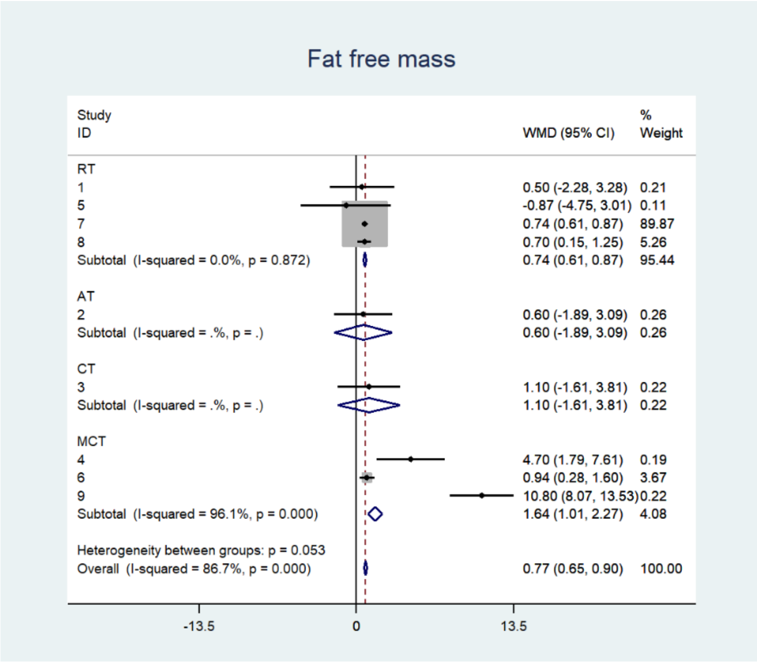

5. 30 s chair stand test

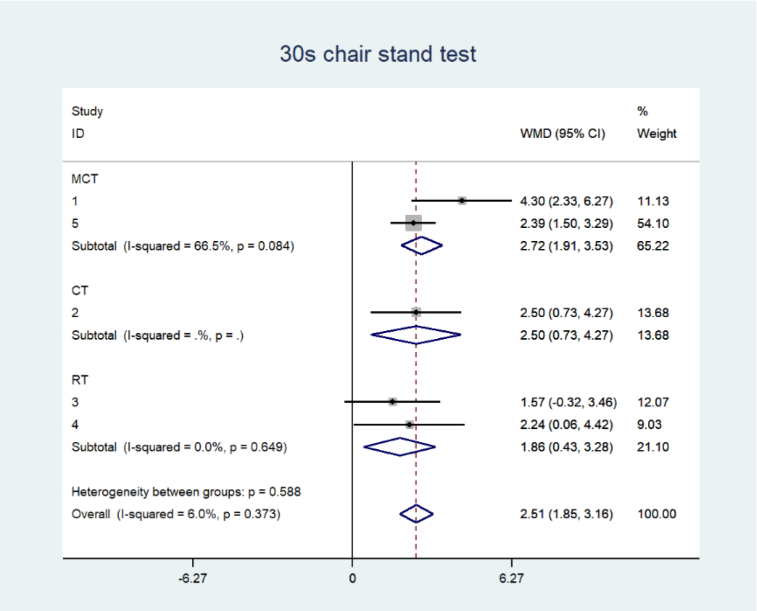

6.Gait speed

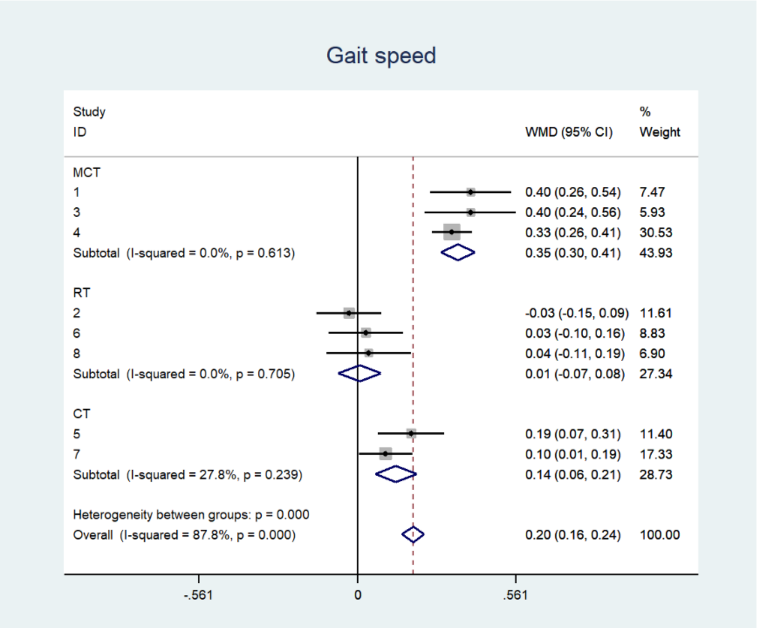

Handgrip strength
(All studies)

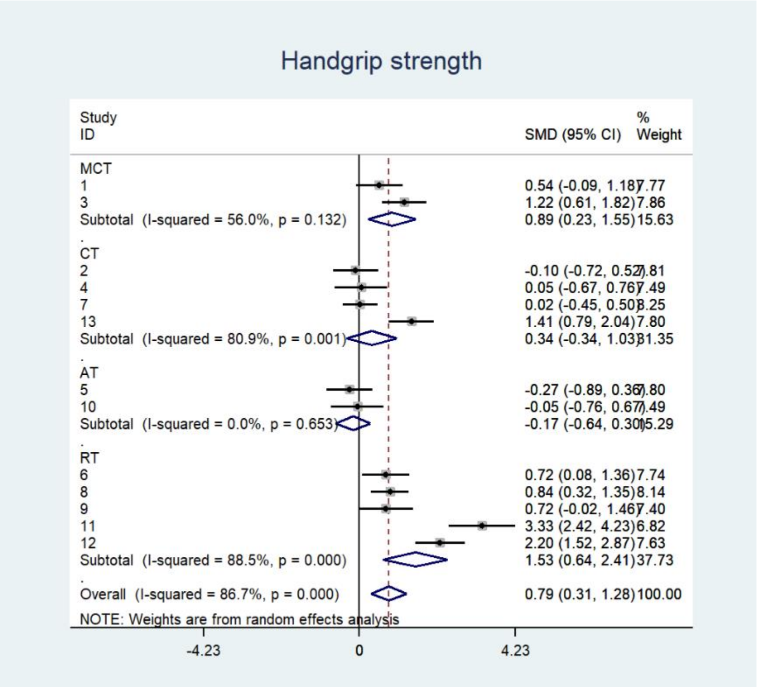

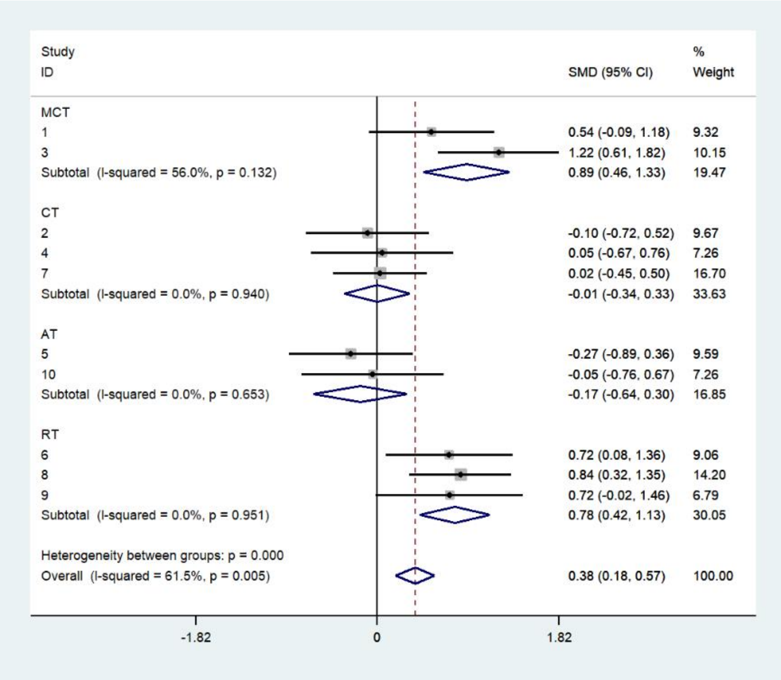

Deleted some data

Note: AT =aerobic training, RT =resistance training, CT = combined resistance with aerobic training, MCT= multiple component training,
